# Dried blood spot omega-3 and omega-6 long chain polyunsaturated fatty acid levels in 7–9 year old Zimbabwean children: a cross sectional study

**DOI:** 10.1186/s12907-016-0035-7

**Published:** 2016-08-05

**Authors:** Grace Mashavave, Patience Kuona, Willard Tinago, Babill Stray-Pedersen, Marshall Munjoma, Cuthbert Musarurwa

**Affiliations:** 1 Department of Chemical Pathology, College of Health Sciences, University of Zimbabwe, PO BOX A178, Avondale, Harare, Zimbabwe; 2 Department of Paediatrics and Child Health, College of Health Sciences, University of Zimbabwe, Harare, Zimbabwe; 3 Department of Community Medicine, College of Health Sciences, University of Zimbabwe, Harare, Zimbabwe; 4 Division of Women and Children, Oslo University Hospital and Institute of Clinical Medicine, University of Oslo, Oslo, Norway; 5 Department of Obstetrics and Gynaecology, College of Health Sciences, University of Zimbabwe, Harare, Zimbabwe

**Keywords:** Omega-3 long chain-polyunsaturated fatty acids, Docosahexaenoic acid, Eicosapentaenoic acid, Docosapentaenoic acid, Arachidonic acid, Dried blood spot, Children, 7–9 year old

## Abstract

**Background:**

Omega-3 long chain-polyunsaturated fatty acids (LC-PUFAs)–docosahexaenoic acid (DHA), docosapentaenoic acid (DPA) and eicosapentaenoic acid (EPA)– and omega-6 LC-PUFA arachidonic acid (ARA), are essential for optimum physical and mental development in children. Prior to this study, the blood omega-3 LC-PUFA levels were unknown in Zimbabwean children, particularly in those aged 7–9 years, despite the documented benefits of LC-PUFAs. Documentation of the LC-PUFA levels in this age group would help determine whether interventions, such as fortification, are necessary. This study aimed to determine dried whole blood spot omega-3 and omega-6 LC-PUFA levels and LC-PUFA reference intervals among a selected group of Zimbabwean children aged 7–9 years old.

**Methods:**

We conducted a cross sectional study from September 2011 to August 2012 on a cohort of peri-urban, Zimbabwean children aged 7–9 years. The children were born to mothers enrolled at late pregnancy into an HIV prevention program between 2002 and 2004. Dried whole blood spots were sampled on butylated hydroxytoluene antioxidant impregnated filter papers and dried. LC-PUFAs were quantified using gas liquid chromatography. Differences in LC-PUFAs between groups were compared using the Kruskal Wallis test and reference intervals determined using non-parametric statistical methods.

**Results:**

LC-PUFAs levels were determined in 297 Zimbabwean children of whom 170 (57.2 %) were girls. The study determined that LC-PUFAs (*wt/wt*) ranges were EPA 0.06–0.55 %, DPA 0.38–1.98 %, DHA 1.13–3.52 %, ARA 5.58–14.64 % and ARA: EPA ratio 15.47–1633.33. Sixteen participants had omega-3 LC-PUFAs levels below the determined reference intervals, while 18 had higher omega-6 LC-PUFAs. The study did not show gender differences in omega-3 and omega-6 LC-PUFAs levels (all *p* > 0.05). EPA was significantly higher in the 8 year age group compared to those aged 7 and 9 years (median; 0.20 vs 0.17 vs 0.18, respectively, *p* = 0.049). ARA: EPA ratio was significantly higher in the 7 year age group compared to those aged 8 and 9 years (median; 64.38 vs 56.43 vs 55.87 respectively, *p* = 0.014).

**Conclusions:**

In this cohort of children, lower EPA levels and higher ARA: EPA ratios were observed compared to those reported in apparently healthy children elsewhere. The high ARA: EPA ratios might increase the vulnerability of these children to inflammatory pathologies. Identification and incorporation into diet of locally produced foodstuffs rich in omega-3 LC-PUFAs is recommended as well as advocating for dietary supplementation with omega-3 fish oils and algae based oils.

## Background

Omega-3 long chain-polyunsaturated fatty acids (LC-PUFAs)—docosahexaenoic acid (DHA), eicosapentaenoic acid (EPA) and docosapentaenoic acid (DPA)—are essential for growth, development and general health [1]. Omega-6 LC-PUFA arachidonic acid (ARA) is essential for brain development [2]. DHA is especially critical for optimal brain [2], cognitive [3] and behavior development. EPA is a precursor of anti-inflammatory eicosanoids (prostaglandins (3 series), leukotrienes (5 series) and thromboxanes (TXA_3_)) [4], and adequate intake of EPA is closely related to positive immunological [5], inflammatory [6] and metabolic [7] outcomes. DPA has been reported to have beneficial effects which include inhibiting platelet aggregation, stimulating endothelial cell migration and regulating gene expression [8]. Though Arachidonic acid (ARA) is a precursor of pro-inflammatory eicosanoids (prostaglandins (2 series), leukotrienes (4 series) and thromboxanes(TXA_2_)) [4]; it produces some metabolites that are required for systemic homeostasis [9]. ARA and its metabolite lipoxin A_4_ have been shown to function as endogenous anti-diabetic molecules [10]. ARA together with LC-PUFAs and their anti-inflammatory products: lipoxins, resolvins, protectins and maresins suppress production of pro-inflammatory eicosanoids, limit inflammation, enhance wound healing, resolve inflammation thus restoring normal cellular, tissue and organ function [10].

Supplementation with omega-3 LC-PUFAs in cardiovascular diseases (CVD) [11], diabetes mellitus [12], hypertension [13], sickle cell anemia [14], inborn errors of metabolism [15, 16], non-alcoholic fatty liver disease [17, 18], attention-defect/hyperactivity disorder [19] autism [20] and asthma [21] has been reported to prevent or alleviate symptoms in children, though other studies have reported conflicting results [22–25].

The above disorders are due to deficiencies in omega-3 fatty acids. Deficiencies in omega-3 fatty acids may result from factors that affect availability of omega-3 LC-PUFAs and influence the metabolism of essential fatty acids (EFA) to LC-PUFAs. These include, imbalances in metabolic pathways [26], genetics [27], imbalances in ARA: EPA ratios [28] and sex hormones [29]. The imbalances in the metabolic pathway may result from linoleic acid (LA) competing with α-linolenic acid (ALA) for the endogenous conversion of ALA to the long chain derivatives EPA and DHA and also inhibition of incorporation of DHA and EPA into tissues [26]. Therefore high levels of LA in the diet result in low ALA and low omega-3 LC-PUFA levels. This in turn affects the omega-6: omega-3 (ARA: EPA) ratios which are critical in human health outcomes [20, 28]. The high levels of LA lead to increased activity of the ARA metabolic pathway [4], which has deleterious effects such as neurological and neurodevelopmental disorders [30]. High concentrations of ARA compete with EPA for incorporation into cell membrane phospholipid leading to high ARA: EPA ratios [28]. The low omega-3 LC-PUFA levels can be due to deficiencies and defects in the Δ6 or Δ5 desaturase enzyme [31] or mutations in the fatty acid desaturase (FADS) gene [27]. Protein malnutrition, carnitine and α-tocopherol enzyme deficiency as well as excess oxygen free radical production in chronic diseases also affect LC-PUFA availability [32]. Oestrogen and testosterone, have been reported to affect EFA metabolism hence availability of long chain metabolites, leading to higher levels in females compared to males [29]. The conversion of EFA into their long chain metabolites is stimulated by oestrogen and inhibited by testosterone [29].

Despite the reported benefits of omega-3 LC-PUFAs in children, most studies demonstrating the nutritional importance of omega-3 LC-PUFAs in children have been carried out in developed countries [2, 3, 11–13], [15–17, 19, 21] with a limited number of studies on African children [14, 33–35]. In most of sub-Saharan Africa healthcare facilities, omega-3 LC-PUFA levels are not on clinical laboratories test menus because the laboratories lack the expertise and technology to perform the tests [36]. In the past the assessment of omega-3 LC-PUFAs has been hindered by difficult methodology [37] and sample instability [38, 39]. Analysis has since been revolutionized by the use of minimally invasive dried blood spots (DBS) [40], which allow the estimation of fatty acid composition of red blood cells and plasma phospholipids that are more reflective of the nutritional status [41]. To date, only a few studies have developed protocols for testing LC-PUFAs in DBS [37–40, 42], with none being carried out in Africa.

In Southern Africa, a region with high prevalence of childhood infectious diseases [43], the levels of omega-3 LC-PUFAs in children are unknown, except in South Africa where the positive effects of omega-3 LC-PUFA supplementation on cognitive development were reported in children aged between 6 and 11 years [33–35]. There is paucity of data on omega-3 LC-PUFA levels in the rest of African countries, including Zimbabwe, especially in children whose adequate intake of LC-PUFAs should be ensured for cognitive development and other positive health outcomes [44].

Monitoring of fatty acid levels and results interpretation in individual patients or in populations require availability of reference intervals obtained from apparently healthy individuals [45]. Fatty acids reference intervals have been established for glycerophospholipids in German children aged 2 and 6 years, for whole blood in apparently healthy European children aged 3–8 years [46] and in apparently healthy Spanish children who were on a normal diet for their age elsewhere [15] but these may not be transferable to a different population. However, scanty studies have been done in low income settings particularly in African children and none in Zimbabwe. At present no reference intervals for LC-PUFAs have been established in these settings.

In this study, the levels of omega-3 LC-PUFAs were determined in Zimbabwean children aged between 7 and 9 years using DBS and reference intervals for LC-PUFAs were determined. The LC-PUFAs were compared between groups by gender and by age.

## Methods

### Study design and setting

From September 2011 to August 2012 we conducted a cross sectional study at three peri-urban primary health care clinics around Harare, the capital city of Zimbabwe. Children aged 7 to 9 years born between 2002 and 2004 to a cohort of mothers previously recruited at late pregnancy from a national HIV prevention of mother to child transmission (PMTCT) program [47], were invited to participate in the study. These children were also eligible if they had no major chronic diseases. Children who were not born to the specified cohort, or who were siblings to the original cohort or whose care givers declined to allow them to take part in the study were excluded as well as those who were HIV-infected, those with inflammatory pathologies and other chronic diseases like diabetes mellitus that may influence the fatty acid composition. All the children were tested for HIV infection at birth, at 6 weeks and subsequently after 18 months.

The study protocol and consent forms were approved by the Joint Research Ethics Committee: (JREC/170/12), and the Medical Research Council of Zimbabwe: (MRCZ/B/359). The consent forms were also approved by the Norwegian Research Ethics Committee. Permission to ship participant samples abroad for laboratory analysis was granted by the Research Council of Zimbabwe. Written informed consent to participate in the study and for long term specimen storage and shipping was obtained from parents or legal guardians and written assent was also obtained from all the children.

### Blood collection

After cleaning, warming and punching a fingertip with an automatic lancing device equipped with a sterile lancet, a drop of non-fasting capillary whole blood sample (~50 μl) from each participant was spotted onto each of the four pre-defined circles on a Whatman 903 (Lot number 6833909/82) filter paper cards (GE Healthcare, UK), which was impregnated with anti-oxidant (butylated hydroxytoluene (BHT; 50 mg/100 ml in ethanol) (Sigma Aldrich Limited, Gillingham Dorset, UK)), for the determination of omega-3 LC-PUFA levels [39]. BHT in Ethanol was prepared at a concentration of 0.05 %. Each spot on the Whatman 903 filter paper card to be used for blood collection was impregnated with the BHT in ethanol solution, by fully covering each collection spot with the solution (by adding two drops). The cards were air-dried for an hour and placed in a sealed polyethylene bag overnight. The filter paper cards were treated with the BHT antioxidant to prevent the polyunsaturated fatty acids losses due to oxidation. The samples were air-dried for 3 h and stored at −25 °C in a zip lock foil paper (Whatman, Maidstone/Banbury UK) with a desiccant (Whatman, Maidstone/Banbury UK) [39].

### Preparation of fatty acid methyl esters (FAMEs) in DBS, extraction, purification and detection

#### Fatty acid extraction and detection

Analysis of PUFAs was carried out at the Nutrition Group Laboratories, Institute of Aquaculture at the University of Stirling, Scotland UK, using a fingertip rapid method that was evaluated by Marangoni et al. [40], and validated by Bell et al. [39]. One DBS circle (per sample), one DBS circle (internal standard), and one DBS circle (control) were cut out from the main DBS filter paper cards using a pair of scissors and forceps and were placed each in a pre-labeled 10 ml screw cap vial which was loaded on the CTC-PAL machine carousel. At the beginning and end of each batch of samples or once a week, a marinol FAME secondary reference material and the Supelco 37 controls were run simultaneously with each run/batch of participants to ensure reproducibility of the known control values. The fatty acids from participants’ samples, standards and quality control samples were transesterified to fatty acid methyl esters (FAMEs) using 1.25 M HCL/Methanol and incubated in dry heating block at 70 °C for 1 h [39]. This was a modification of the one step direct transmethylation method described by Lepage and Roy [48]. The FAMEs were extracted into iso-hexane (Fisher Scientific, Loughborough, UK) and precipitated with saturated potassium chloride (Fisher Scientific, Loughborough, UK) before purification [39]. Purification was done by passing the extract through a preconditioned solid phase extraction (SPE) cartridge (Clean-Up Extraction Columns, UCT, Bristol, USA) (prewashed with iso-hexane) and eluted with iso-hexane—diethyl ether (95:5 *v/v)* (Fisher Scientific, Loughborough, UK) [39]. The FAME extracts were dried under nitrogen (BOC Gases, Guildford, UK) using a nitrogen evaporator (N-EVAP™ 111, Organomation Associates, Berlin USA) at room temperature, re-dissolved in 0.2 ml iso-hexane and placed in an autosampler vial (Chromacol, Herts, UK) prior to gas liquid chromatography (GLC) (Thermo Fisher Trace, Hertfordshire UK) analysis [39]. The stability of the fatty acid analytes in whole wet blood and in the dried whole blood samples at different temperatures (room temperature, 4 and −20 °C) and at different times periods ((3 h post drying) and subsequent samples were removed for analysis after 48 h, 7 days, 14 days and 28 days in storage) was investigated by Bell et al. [39].

The GLC was calibrated using duplicate injections of standard mixtures of known composition (Supelco AOCS No. 37 standards containing 14:0 to 22:6n-3). A second standard, a Supelco custom mix containing 14:0, 16:0, 16:In-7, 18:0, 18:In-7, 18:2n-6, 10:0, 10:5n-3, 22:In-9, 22:5n-3 and 22-6n-3, was used to check calibration and replication by making three consecutive analyses. FAMEs (1 μl) were injected, separated and quantified by GLC using a 60 m × 0.32 mm × 0.25 μm film thick capillary column (ZB Wax; Phenomenex, Macclesfield, Cheshire, UK). Hydrogen gas (BOC Gases, Guildford, UK) was used as a carrier gas at a flow rate of 4.0 ml/min and the temperature program was from 50 to 150 °C at 40 °C/min then to 195 °C at 2 °C/min and finally to 215 °C at 0.5 °C/min [39]. The FAMEs were detected by a flame ionization detector. The FAMEs were compared to well-characterized in-house standards as well as commercial FAME mixtures (Supelco™ 37 FAME mix; Sigma Aldrich Limited, Gillingham Dorset, UK). Fatty acids ranging from C14:0 to C22:6 carbons were detected. The fatty acid data was collected from the GLC and processed using the Chromocard software computer package for Windows (version 2.1) (Thermoquest Italia S. p. A., Milan, Italy). The FAMEs results were expressed as percent weight of individual fatty acids in total fatty acids (% weight/weight (% *wt/wt*)). Precision and coefficient of variation were done by Bell et al. using the same analytical method, on same instrumentation and in the same establishment as the work published in this manuscript.

The total omega-6 LC-PUFAs measured included; Dihomo-gamma-linolenic acid (DGLA), ARA and Docosapentaenoic acid (DPA) (n-6) whereas the total omega-3 LC-PUFAs included; EPA, DPA (n-3) and DHA. Omega-6 LC-PUFAs were included in the results since they compete with omega-3 LC-PUFAs for the same enzymes for desaturation. The results of EPA, DPA (n-3), DHA, ARA, and the calculated total omega-3 PUFAs, total omega-3 LC-PUFA: total omega LC-PUFAs, ARA: EPA, total omega-6 LC-PUFA: total omega-3 LC-PUFA ratios were selected for data analysis.

### Statistical analysis

Participants’ characteristics were summarized using percentages for categorical variables and median [inter-quartile ranges (IQR)] for continuous non-normal variables. Differences in median omega-3 LC-PUFAs by age group and by gender were compared using the Kruskal-Wallis test. To account for multiple comparisons, we applied the Bonferroni correction which lowers (adjust) the α value (type1 error) to account for the number of comparisons being performed simultaneously, thereby avoiding a lot of spurious positives. Non-parametric statistical methods from Reference Value Advisor v1.3 [49] were used to determine the central 95 % reference interval limits, with the lower limit defined as the 2.5 percentile and the upper limit defined as the 97.5 percentile, together with the 90 % confidence intervals (CI) of the distribution of omega-3 LC-PUFAs. For all statistical comparisons α (*p* value) was set at 0.05. Data entry and analysis was conducted using Statistical Package for Social Scientists (SPSS, New York, USA).

## Results

A total of 319 of the available children from the original cohort participated in the study. Of these children, one had a DBS collected on a non BHT treated filter paper and 21 (6.6 %) were HIV-infected. Those who were HIV infected were all excluded from further analysis. Of the remaining 297, the median age (range) was 9 (7–9) years and 170 (57.2 %) were girls (Table 1).

**Table 1:** Age and gender of the children

Variable	Frequency *n* = 297
Gender of the children	
Boys	127 (42.8 %)
Girls	170 (57.2 %)
Children age group (years)	
7	21 (7.1 %)
8	93 (31.3 %)
9	183 (61.6 %)

*p-values* calculated using Bonferroni test. Statistically Significance (*p* < 0.05) (2-tailed)

### Distribution and comparison of LC-PUFA levels

Sixteen (5.39 %) of the children had EPA, DPAn-3, DHA levels that were below the determined reference intervals and 18 (6.06 %) had ARA levels, ARA: EPA and total omega-6 PUFA: Total omega-3 PUFA ratios that were above the determined reference intervals. The LC-PUFAs (% *wt/wt*) ranges were as follows: EPA 0.06–0.55 %, DPA 0.38–1.98 %, DHA 1.13–3.52 %, ARA 5.58–14.64 % and ARA: EPA ratio 15.47–1633.33 (Table 2).

**Table 2:** Distribution of median (IQR) LC-PUFA levels (% *wt/wt*) by gender and age group of 297 Zimbabwean children

	Variables	LC-PUFAs (% *wt/wt*)							
		EPA	DPA	DHA	ARA	Total Omega-3 LC-PUFA	% Omega LC-PUFA: Total LC-PUFA	ARA: EPA	Total n-6 PUFA: Total n-3 PUFA
	Median (IQR) (*n* = 297)	0.18 (0.15–0.23)	0.79 (0.70-0.89)	2.14 (1.87-2.42)	10.62 (9.77–11.38)	3.13 (2.83–3.49)	18.42 (17.10–19.92)	57.47 (44.72–72.24)	10.82 (9.83–11.79)
	Mean (SD) (*n* = 297)	0.20 (0.071)	0.81 (0.17)	2.15 (0.40)	10.56 (1.26)	3.55 (0.53)	18.11 (2.27)	61.31 (23.64)	10.91 (1.62)
	Range All (*n* = 297)	0.06–0.55	0.38–0.198	1.13–3.52	5.58–14.64	1.73–5.95	13.34–28.11	15.47–163-33	5.94–16.03
Children’s gender median IQR)	Boys (*n* = 127)	0.19 (0.15–0.23)	0.81 (0.72–0.91)	2.11 (1.91–2.46)	10.55 (9.80–11.32)	3.25 (2.62–3.42)	18.38 (17.32–19.94)	57.16 (45.24–71.82)	10.71 (9.78–11.68)
	Girls (*n* = 170)	0.18 (0.15–0.24)	0.78 (0.70–0.89)	2.15 (1.84–2.36)	10.67 (9.73–11.46)	3.10 (2.82–3.47)	18.44 (16.94–19.89)	57.72 (44.09–72.91)	10.95 (9.88–11.97)
Children’s Age group median (IQR)	(7) (*n* = 21)	0.17 (0.13–0.18)	0.78 (0.74–0.86)	2.27 (2.03–2.48)	10.89 (10.68–11.75)	3.18 (2.92–3.53)	18.20 (16.69–19.52)	64.38 (59.71–91.04)	10.60 (9.93–11.89)
	(8) (*n* = 93)	0.20 (0.16–0.24)	0.81 (0.70–0.93)	2.16 (1.93–2.51)	10.63 (9.61–11.39)	3.18 (2.87–3.57)	18.64 (17.35–20.18)	56.43 (41.39–72.02)	10.55 (9.68–11.45)
	(9) (*n* = 183)	0.18 (0.15–0.23)	0.79 (0.70–0.89)	2.10 (1.83–2.35)	10.48 (9.76–11.35)	3.09 (2.80–3.40)	18.34 (16.94–19.88)	55.87 (44.90–70.64)	10.94 (9.87-12.01)
*p-values*		*P* = 0 .049^a^						*P* = .014^a^	

*p-values* calculated using Kruskal Wallis Test Asterisks: Significance tests: ^a^Statistically Significance (*p* < 0.05) (2-tailed)

The median LC-PUFA levels between boys and girls were not significantly different (*p* > 0.05).

**Fig. 1:**
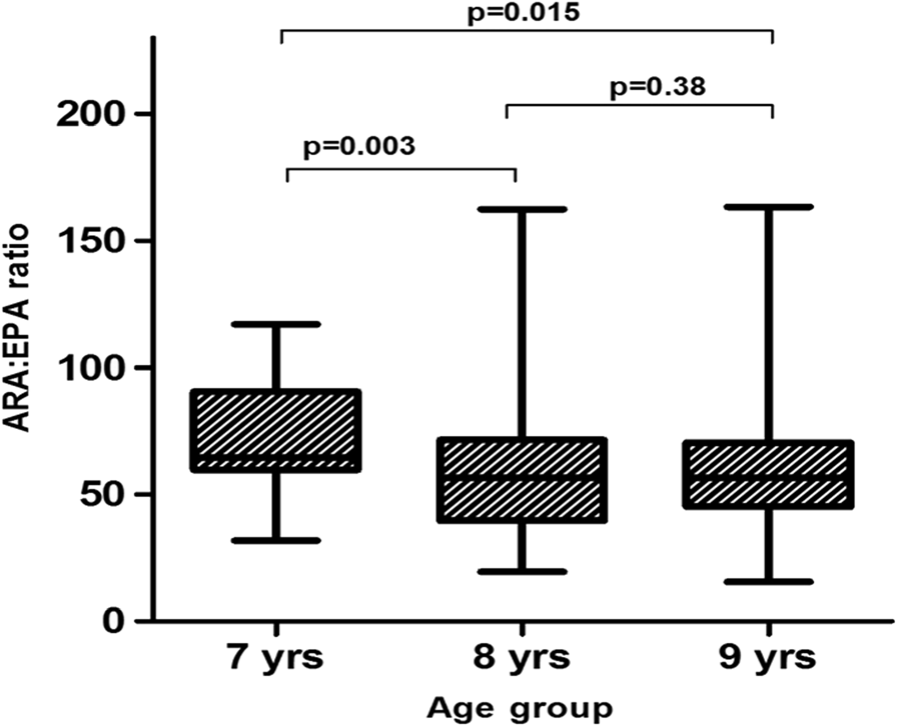
ARA: EPA ratio between ages groups of 297 Zimbabwean children p-values calculated using Bonferroni test. Statistically Significance (p<0.05) (2-tailed)

EPA levels were significantly elevated in the 8 year age group compared to those aged 7 and 9 years (0.17 vs 0.20 vs 0.18, respectively, *p* = 0.049). The median ARA: EPA ratio was significantly elevated in the 7 year age group compared to the other age groups (*p* = 0.014) (Table 2). Distribution of median ARA: EPA ratio was significantly different between age groups after being corrected using the Bonferoni test (Fig. 1).

### Reference intervals of LC-PUFAs

Reference intervals were determined for the 297 7–9 year old Zimbabwean children. Five children (1.7 %) had EPA levels below the 2.5 percentile (0.09 % *wt/wt*), while 6 (2.0 %) had EPA levels above the 97.5 percentile (0.37 % *wt/wt*). Five children (1.7 %) had DPA levels below the 2.5 percentile (0.53 % *wt/wt*) and 6 (2.0 %) were above the 97.5 percentile (1.15 % *wt/wt*). Six children (2.0 %) had DHA levels below the 2.5 percentile (1.35 % *wt/wt*) and 6 (2.0 %) were above the 97.5 percentile (2.93 % *wt/wt*). Six children (2.0 %) had ARA levels below the 2.5 percentile (7.85 % *wt/wt*) and 7 (2.4 %) were above the 97.5 percentile (12.92 % *wt/wt*). The LC-PUFA reference intervals with 90 % CI as determined from the results of the 7–9 year old Zimbabwean children are shown in Table 3.

**Table 3:** Reference Intervals of LC-PUFAs (% *wt/wt*) in Zimbabwean children aged 7–9 years

LC-PUFAs (% *wt/wt*)	Median (IQR) (*n* = 297)	Reference Intervals 7–9 years old *n* = 297	
		2.5th Percentile (90 % CI)	97.5th Percentile (90 % CI)
Omega-3 fatty acids			
ALA (18:3n-3)	0.38 (0.29–0.47)	0.17 (0.16 0.20)	0.73 (0.67 0.86)
EPA (20:5n-3)	0.18 (0.15–0.23)	0.09 (0.08 0.10)	0.37 (0.33 0.45)
DPA (22:5n-3)	0.79 (0.70–0.89)	0.53 (0.49 0.57)	1.15 (1.07 1.26)
DHA (22:6n-3)	2.14 (1.87–2.42)	1.35 (1.26 1.46)	2.93 (2.86 3.16)
Total n-3 PUFA	3.55 (3.22–3.87)	2.55 (2.39 2.64)	4.64 (4.45 5.10)
Omega-6 fatty acids			
ARA (20:4n-6)	10.62 (9.77–11.38)	7.85 (6.86 8.32)	12.92 (12.57 13.55)
Total n-6 PUFA	38.10 (36.72–39.39)	33.18 (31.72 33.91)	41.74 (41.14 42.41)
Other fatty acids			
Total saturated	38.55 (37.45–39.74)	35.82 (35.51 36.12)	42. 71 (41.50 43.18)
Total monounsaturated	16.20 (15.33–17.61)	13.75 (13.37 14.07)	21.39 (20.23 22.04)
Total DMA	3.29 (3.00–3.62)	2.44 (1.99 2.62)	4.15 (3.96 4.38)
ARA: EPA	57.47 (44.72–72.24)	26.51 (21.58 28.26)	110.83 (104.50 144.63)
% n-3LC-PUFA: Total LC-PUFA	18.42 (17.10 19.92)	14.54 (14.06 14.98)	24.56 (23.00 26.34)
Total n-3 LC-PUFA	3.13 (2.83–3.49)	2.14 (1.98 2.28)	4.29 (4.03 4.71)
Total n-6 PUFA: Total n-3 PUFA ratio	10.82 (9.83–11.79)	7.91 (7.42 8.48)	14.48 (14.04 15.13)

Fatty Acids Acronyms: *ALA* “α-Linolenic acid”, *EPA* “Eicosapentaenoic acid”, *DPA* “Docosapentaenoic acid”, *ARA* Arachidonic acid”, *DMA* Dimethylacetal

## Discussion

To our knowledge, this is the first study to report blood levels of omega-3 and omega-6 LC-PUFAs and to determine LC-PUFA reference intervals in 7–9 year old Zimbabwean children.

The levels for omega-3 LC-PUFAs (EPA, DPAn-3 and DHA) of the children in this study were strikingly low, while those of omega-6 LC-PUFA (ARA) were surprisingly high compared to the determined reference intervals and to the results obtained from a UK study on similar age groups and biomarker [42] (EPA 0.20 v 0.56, DPAn-3 0.81 v 1.03, DHA 2.15 v 1.9, ARA 10.56 v 8.17). Generally, these children had very low omega-3 PUFAs and very high saturated fats, monounsaturated and omega-6 fatty acids. The essential omega-3 fatty acid, α-linolenic acid, which is the precursor of the omega-3 LC-PUFAs (EPA, DPA and DHA), mainly found in seeds, nuts and some vegetable oils, was also low in the children under study (median level 0.38 % *wt/wt*) compared to results obtained from a study on similar age groups and biomarker [42]. The highest EPA value obtained in this study of 0.55 % *wt/wt* was lower than the mean values obtained from a study on similar age groups and biomarker [42]. Results of the present study also demonstrated the lowest EPA value of 0.06 % *wt/wt* reported in apparently healthy children compared to results obtained from a study on similar age groups and biomarker [42]. This might be a reflection of the different geographical backgrounds, diet and genetic make-up of the children in the different studies.

The low EPA and high ARA levels are of health concern because they lead to very high ARA: EPA ratios, which are pro-inflammatory, and to very high total omega-6 PUFA: total omega-3 PUFA ratios [30]. The high ratios observed in this study reflect possible imbalances in the dietary intake of omega-6 and omega-3 rich foods. The imbalances could be as a result of contemporary changes in human nutrition caused by increased consumption of diets rich in saturated fats (rich in beef), monounsaturated and omega-6 fatty acids including the use of cooking oils, vegetable oils and bread spreads rich in omega-6 PUFAs, accompanied by a decreased intake of omega-3 PUFA-rich foods [50]. Deficiencies in DHA exposes children between the ages of 7 and 9 to impaired brain development during the 7–9 year old “Brain Spurt” [44], possibly leading to compromised intellectual development, academic performance, low verbal learning ability, memory and learning difficulties [33, 34].

The LC-PUFA levels of all parameters except DHA were lower in the present study compared to the expected values from the University of Stirling Aquaculture laboratory [39] that used the same method of analysis and sample type (DBS) as the present study (EPA 0.20 v 0.91, DPA 0.81 v 2.47, DHA 2.15 v 2.47, ARA 10.56 v 13.88). The median EPA level in the present study was similar to that obtained by Mohammed et al. on pregnant Zimbabwean black women [36], indicating a general view of the dietary intake of foods poor in omega-3 LC-PUFAs and α-linolenic acid in the population.

Our findings of no differences by gender in median LC-PUFAs levels were in agreement with those of Glaser et al. on a paediatric population [45]. However, another study on a paediatric population reported a more pronounced low omega-3 and omega-6 LC-PUFA status in girls than boys [42], while another study reported slightly higher omega-6 ARA in boys than in girls [46]. Yet another study, reported that sex hormones (testosterone and oestrogen) influence the enzymatic synthesis of LC-PUFAs, leading to gender related differences in LC-PUFA status with higher levels occurring in adult females [29]. The reason for the lack of gender differences in LC-PUFA levels observed in this study was perhaps due to the younger age of the participants.

The observed differences in median EPA and ARA: EPA ratio across and between the children’s age groups is probably due to differences in dietary content. The 7 year old children had lower EPA and higher ARA values leading to high ARA: EPA ratio. The low EPA values observed in the children understudy are however constrained by the lower sample size in this particular age group; hence the results should be interpreted with caution. These findings were similar to UK study on similar age groups and biomarker [42] and also similar to the study with European children though with no age dependence for ARA [46]. An Italian study with differences in fatty acids by age groups concluded that the differences resulted either from lower intakes or the rates of utilization and resulting physiological requirements which are higher in younger age groups compared to older age groups [51].

The study also determined DBS LC-PUFA reference intervals for the apparently healthy 7–9 year old Zimbabwean children. However, these DBS LC-PUFA reference intervals cannot be generalised to the rest of the population since the LC-PUFA results were from children from a select group born to a cohort residing in a peri-urban setting, which did not include rural and urban children. The determined LC-PUFA reference intervals were not comparable to those of three other studies which determined LC-PUFA reference intervals perhaps due to methodological differences [15, 45, 46].

Our results showed generally low values across the omega-3 LC-PUFA range. The levels of these LC-PUFAs could be improved by identifying and encouraging the intake of locally available omega-3 LC-PUFA rich foods. Supplementation with EPA and DHA omega-3 fish oils and algae based oils to balance ARA levels is recommended in the children since low omega-3 LC-PUFA levels are recognized confounders of general health. Limited intake of ARA-rich foods is also recommended if the desirable total omega-6 PUFA: total omega-3 PUFA ratio of 1–4:1 [6] is to be achieved. There is need for a public awareness campaign on food sources rich in omega-3 LC-PUFAs and the benefits of omega-3 LC-PUFAs throughout life. We recommend further studies on children under the age of 5 years and inclusion of children from rural and urban Zimbabwe to ascertain their omega-3 LC-PUFA levels. Results from such studies could be used as the basis for establishing reference intervals that can be generalized to the whole Zimbabwean paediatric population, as well as the basis for food fortification and the implementation of omega-3 LC-PUFA supplementation policies.

The study has a number of limitations. Firstly no dietary intake assessment was done during specimen collection to ascertain the practices that could explain the low omega-3 LC-PUFA levels, hence, the causes of low omega-3 LC-PUFA levels are assumption based. Secondly, the determined reference intervals are limited to the children born to the specified cohort as a limited age group was used for this study. The study population was also restricted to children in a peri-urban setting that may not be truly reflective of the Zimbabwean population. The determined DBS reference intervals could also not be compared to those from other populations because of analytical method differences [15, 45, 46]. Lastly, the three age groups were unequal and this could distort the distribution of omega-3 LC-PUFAs findings by age.

## Conclusion

Nevertheless, this is an important study that observed very low EPA levels and very high ARA: EPA and total omega-6 PUFA: total omega-3 PUFA ratios ever reported in apparently healthy children. The findings of this research could be the basis for future omega-3 LC-PUFA intervention studies in Zimbabwe. The techniques learnt for LC-PUFA analysis could the basis of technology transfer to Zimbabwe.

## Abbreviations

% wt/wt, % weight to weight; ALA, α-linolenic acid; ARA, arachidonic acid; BHT, butylated hydroxytoluene; CI, confidence intervals; CVD, cardiovascular diseases; DBS, dried blood spot; DGLA, dihomo-gamma-linolenic acid; DHA, docosahexaenoic acid; DPA, docosapentaenoic acid; EFA, essential fatty acids; EPA, eicosapentaenoic acid; FADS, fatty acid desaturase; FAME, fatty acid methyl ester; GLC, gas liquid chromatography; IQR, inter-quartile ranges; LA, linoleic acid; LC-PUFA, long chain polyunsaturated fatty acids; PMTCT, prevention of mother to child transmission; SPE, solid phase extraction; TXA_2_, thromboxanes; TXA_3_, thromboxanes
